# Virtual screening, molecular dynamics and binding energy-MM-PBSA studies of natural compounds to identify potential EcR inhibitors against *Bemisia tabaci* Gennadius

**DOI:** 10.1371/journal.pone.0261545

**Published:** 2022-01-21

**Authors:** Harmilan Kaur Mangat, Manisha Rani, Rajesh Kumar Pathak, Inderjit Singh Yadav, Divya Utreja, Pardeep Kumar Chhuneja, Parveen Chhuneja

**Affiliations:** 1 School of Agricultural Biotechnology, Punjab Agricultural University, Ludhiana, India; 2 Department of Chemistry, Punjab Agricultural University, Ludhiana, India; 3 Department of Entomology, Punjab Agricultural University, Ludhiana, India; National Agri-Food Biotechnology Institute (NABI) Mohali, INDIA

## Abstract

Whitefly (*Bemisia tabaci* Gennadius) is a hemipteran phyto polyphagous sucking insect pest which is an important pest of cotton that causes economic losses to the crop by reducing its yield and quality. Ecdysteroids such as 20-hydroxy ecdysone (20-E), play a significant role in larval moulting, development, and reproduction in pterygota insects. Receptor of 20-E, that is Ecdysone Receptor (BtEcR) of *Bemisia tabaci* has been targeted to prevent fundamental developmental processes. To identify potent inhibitors of BtEcr, 98,072 natural compounds were retrieved from ZINC database. A structure-based virtual screening of these compounds was performed for evaluating their binding affinity to BtEcR, and top two compounds (ZINC08952607 and ZINC04264850) selected based on lowest binding energy. Molecular dynamics simulation (MDS) study was performed for analyzing the dynamics and stability of BtEcR and top-scoring ligand-BtEcR complexes at 50 ns. Besides, g_mmpbsa tool was also used to calculate and analyse the binding free energy of BtEcR-ligand complexes. Compounds ZINC08952607 and ZINC04264850 had shown a binding free energy of −170.156 kJ mol^-1^ and −200.349 kJ mol^-1^ in complex with BtEcR respectively. Thus, these compounds can be utilized as lead for the development of environmentally safe insecticides against the whitefly.

## 1. Introduction

Cotton (*Gossypium spp*.), popularly known as ’White Gold’, is one of the world’s most valuable commercial fiber and oil crops. It has a significant role in the industrial and agricultural economy. In India, it plays important role in the national economy and provides employment to more than eight million people. The main bottleneck in cotton cultivation is the yield losses due to biotic stresses such as insect-pests’ attacks, diseases, and crop-weed competition. Major insect-pests of cotton include bollworms, foliage feeders, and sucking pests, which cause serious damage to different parts of the plant through the cropping season. With the introduction of Bt cultivars of cotton having resistance against cotton bollworms, sucking pests especially the whitefly, have emerged as serious problems (https://atariz1.icar.gov.in/pdf/Report-KVKs-at-Farmers-Doorsteps.pdf).

The whitefly (*Bemisia tabaci* Gennadius) is a polyphagous insect that feeds on more than 500 plant species including field crops, vegetables, fruits, oil seed crops, ornamental plants, etc. It is a highly destructive pest for crops, and also acts as a vector for several viral pathogens in cultivated plants [1–3].

On cotton crops, the damage caused by whitefly is due to direct feeding and through lint contamination by the development of sooty mould honeydew on its execrate [4]. The whitefly is also the vector of Cotton leaf curl virus (CLCuV). Due to a lack of effective genetic resistance, farmers occasionally had to rely on insecticides for efficient management of whitefly. However, in certain situations, a highly effective integrated pest management (IPM) strategy has been developed, disseminated and ensured its adoption that has given effective control of the pest. Supervised use of chemical insecticides using economic threshold level (ETL) is one component under IPM [5].

Several types of insecticides such as pyrethroids, organophosphates and neonicotinoids are approved for this pest but *B*. *tabaci* has developed resistance against these insecticides [6–9]. Besides, these chemical insecticides are also hazardous for the environment and injurious to other non-target living organisms [5]. So there is a need of natural insecticides that must possess selective insecticidal action against *B*. *tabaci*.

In whitefly and other arthropods, the external cuticle is rigid and inexpansible. Therefore, these animals develop through a moulting process that facilitates growth and required morphological changes. To enable the co-ordination of growth with environmental signals, moulting takes place under the regulation of steroid hormones such as ecdysteroids -a-ecdysone (aE) and its biologically active form i.e. 20-hydroxy ecdysone (20-E). Ecdysone Receptor (EcR) regulates larval moulting, metamorphosis, and reproduction process [10, 11].

Ecdysone receptor is a ligand-dependent transcription factor [12, 13] that belongs to the superfamily of nuclear receptors [10]. Ecdysone receptor is found only in invertebrates and serves as a key regulator of the gene expression during the development and reproduction of most invertebrates [11]. The functional unit of the receptor is a noncovalent heterodimeric complex of two proteins i.e. Ecdysone receptor (EcR) and another nuclear receptor family member, Ultraspiracle Protein (USP) [10, 14–17]. EcR and USP have a similar modular domain structure as that of all other nuclear receptors, constituting a well-conserved DNA binding domain (DBD) and a conserved ligand-binding domain (LBD) [14]. The Ecdysone binding site is present in the LBD of the EcR subunit, but USP must be dimerised with EcR for high-affinity binding of ligand. EcRs heterodimerize with USPs to attain transcriptional activity in the presence of ecdysteroids [18]. EcR can be used as a potential target for the searching and designing of environmentally safe insecticides that can specifically inhibit the molting process of insects.

Structure-based virtual screening was used for screening of potential inhibitors that can interact with EcR of *B*. *tabaci*. In our study, we performed compound searching and retrieval, virtual screening, molecular dynamics simulation (MDS), and binding free energy analysis of natural compounds stored in ZINC database [19, 20] to identify potential inhibitors against EcR target of *B*. *tabaci*.

## 2. Materials and methods

### 2.1. Retrieval and preparation of target protein structure

The structure of *B*. *tabaci* ecdysone receptor (BtEcR) (PDB ID: 1Z5X) was retrieved from RCSB-Protein Data Bank (https://www.rcsb.org/) [21]. Target protein was prepared using Autodock tool [22]. Hydrogen atoms were added, and a partial atomic charge (Kollman charge) was assigned to the BtEcR structure. Grid box was generated by choosing co-crystallized bound ligand area to define the binding site for virtual screening based molecular docking. The prepared protein structure was saved in.pdbqt format.

### 2.2. Retrieval and preparation of ligand structures

The natural compounds’ subset was downloaded from the ZINC database (https://zinc.docking.org/). The 98,072 natural compounds were retrieved in the.sdf file format. Openbabel tool (http://openbabel.org/wiki/Main_Page) was used to convert sdf files to pdb file format. Further, a python script was used to prepare pdbqt file of selected natural compounds for the purpose of docking study with BtEcR target using AutoDock vina [23].

### 2.3. Structure based virtual screening

Virtual screening is a lead discovery technique that plays an important role in the discovery of potential compounds. It can screen large number of compounds using the residues present in the active site of a target. It enables the screening of large data sets to find the structures possessing the tendency to bind with the target. AutoDock vina [23] is a molecular docking tool which determines the preferred relative orientation of ligand during its docking on target protein, and form a stable complex with lowest binding energy. In this study, the natural compounds were screened against the BtEcR target, and compounds with favourable binding energy were selected. PyMol (https://pymol.org/2/) was used to generate these ligand-protein complex structures, and Ligplot was used to visualise them [24].

### 2.4. Physicochemical properties prediction

Drug-likeliness and physico-chemical properties of 10 top scoring natural ligands were retrieved from ZINC database and the remaining unknown properties were calculated using MarvinSketch software (http://www.chemaxon.com/products/marvin/marvinsketch). Total eight parameters were estimated, and these include molecular weight (MW), logP, hydrogen-bond donor, hydrogen-bond acceptor, polar surface area (2D), polarizability, Van der Waal’s surface area (3D), and refractivity [25].

### 2.5. Molecular Dynamics Simulation (MDS)

GROMACS 2018.1 was used for molecular dynamics analysis and stability of BtEcR and top-scoring ligand-BtEcR complexes. Three systems were generated and subjected to 50 ns MDS studies, one for estimating the stability of the BtEcR and other two for BtEcR-ligand complexes. The stability of the selected protein-ligand complex was analysed in the presence of a solvent. All three systems were solvated in a cubic box using a simple point charge model. Ligand topology was generated using the ProDRG server [26]. Protein topology was generated using the GROMOS 9653a6 force field [27]. The systems were neutralized by adding 8 Na^+^ ions. Steepest energy minimization was carried out for all the systems to obtain the maximum force below 1000 kJ mol^-1^ nm^-1^ to eliminate the steric hindrances. Particle Mesh Ewald method was used to calculate long-range electrostatic interactions [28]. Radius cut-off of 1.0 nm was used for the computation of Lennard–Jones, and Coulomb interactions. For constraining the H-bond lengths, the LINCS algorithm [29] was used. The time step was maintained at 2 fs for the sampling of simulation trajectory. 10 Å Cut-off distance of 10 Å was used to predict the short-range non-bonded interaction, while for long-range electrostatics, the PME method was used with 1.6 Å Fourier grid spacing. All bonds including H-bond were fixed by the Shake algorithm [30]. The systems were stabilized after energy minimization. Then position restraint simulation was carried out under NVT and NPT conditions for maintaining the volume, temperature, and pressure. Root-mean-square deviation (RMSD), root-mean-square fluctuation (RMSF), radius of gyration (Rg) and H-bonds were calculated by gmx rms, gmx rmsf, gmx gyrate and gmx hbond. Principal component analysis (PCA) was done by gmx covar and gmx anaeig. Finally, xmgrace was used for the generation and visualization of the plots [31].

### 2.6. Binding energy calculation through MM-PBSA

Binding free energy of protein-ligand complexes (BtEcR-ZINC08952607, and BtEcR- ZINC04264850) was calculated using the g_mmpbsa tool [32]. g_mmpbsa calculated the binding free energy of complex structure using the Molecular Mechanics Poisson–Boltzmann surface area (MM-PBSA) method. The potential energy of solvation (electrostatic + van der Waals interaction) and the free energy of solvation (polar + non-polar solvation energies) were determined for last 5 ns of molecular dynamics simulation trajectory.

## 3. Results and discussion

### 3.1. Structural analysis, visualization and generation of grid box for virtual screening

Autodock tools was used for analysis and visualization of BtEcR structure. It is a well known tools for analysis of macromolecular structure, molecular docking, and deciphering the key amino acid residues of receptor involved in interaction with small molecules [23]. The grid box of BtEcR was generated with centre X = 25.114, Y = 72.57, Z = 4.445, and size X = 46, Y = 48, Z = 44 by AutoDock tool and define in a configuration file, besides; the energy range and exhaustiveness were kept 4 and 8, respectively. The generated grid box size defined in configuration file was further utilized to carry out interaction studies of BtEcR with natural compounds through virtual screening.

### 3.2. Identification of natural lead ligand through structure-based virtual screening

For structure-based virtual screening, a subset of natural compounds (n = 98,072) was downloaded from the ZINC database and the structure of target protein- BtEcR from RCSB PDB. All the ligands and target were prepared using Autodock tools. The pdbqt file of BtEcR and all the selected natural ligands were subjected to virtual screening based molecular docking by AutoDock vina using python script available at vina website. The top 10 ligands were selected based on lowest binding energy which ranged between −13.4 Kcal/mol and −12.9 Kcal/mol.

### 3.3. Identification and analysis of key residues stabilizing protein-ligand complex

**Fig 1:**
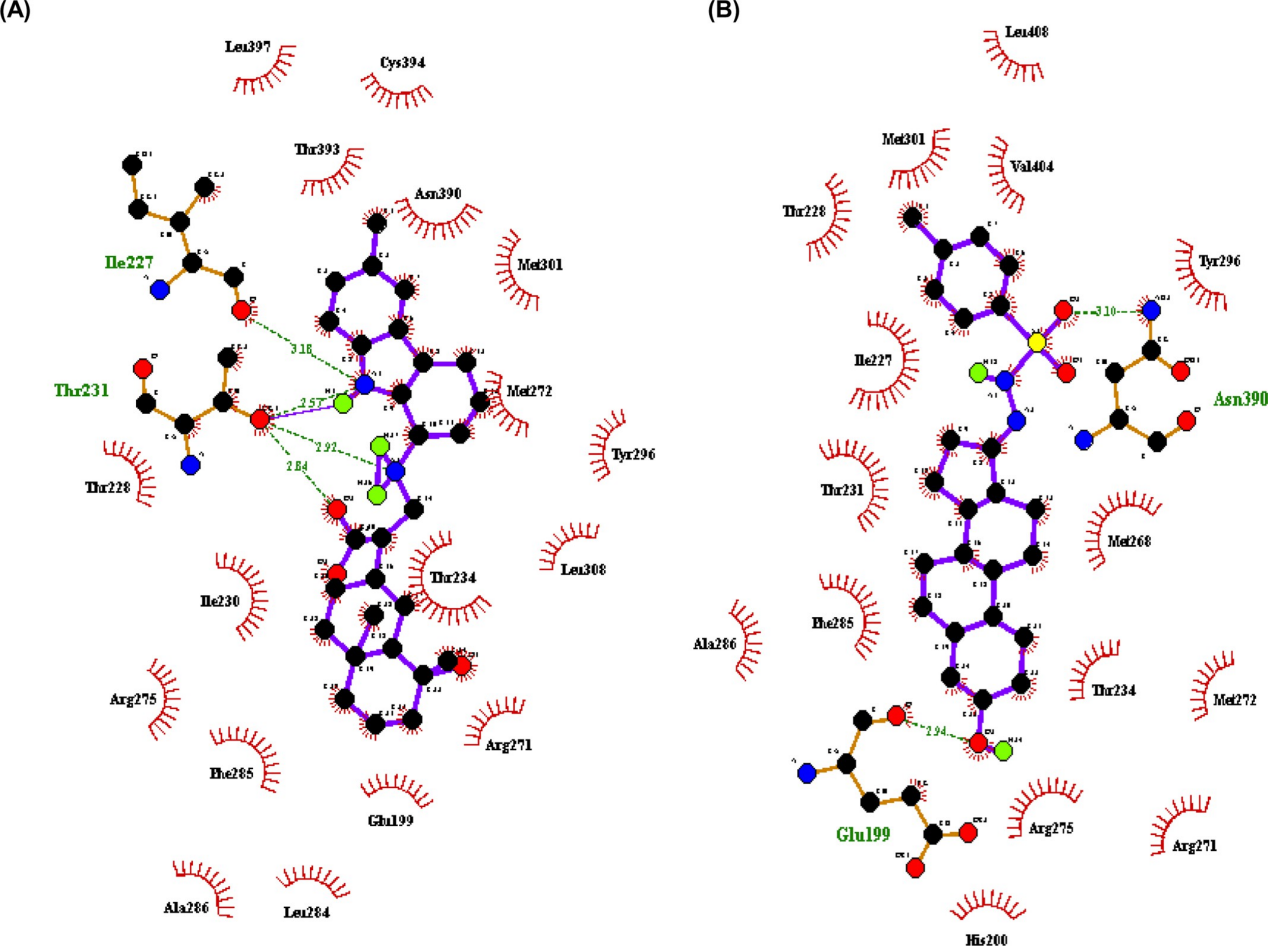
Protein-ligand interaction analysis. (A) BtEcR-ZINC08952607 docked complex structure generated through Ligplot showing hydrogen bonds with amino acid residues Thr231 and Ile227 as green lines and hydrophobic interactions as red arcs. (B) BtEcR-ZINC04264850 docked complex structure generated through Ligplot showing hydrogen bonds with amino acid residues Glu199 and Asn390 as green lines and hydrophobic interactions as red arcs.

Various interactions such as H-bond, hydrophobic interactions, and the amino acid residues involved in H-bonding of these complexes were then visualized using Ligplot and are given in Table 1. Both top two natural ligands ZINC08952607 and ZINC04264850, have binding affinity of -13.4 Kcal/mol. The ZINC08952607 forms four hydrogen bonds with EcR, three with Thr231 having bond length 2.84 Å, 2.92 Å, and 2.57 Å and one with Ile227 having bond length 3.18 Å. However, ZINC04264850 forms two hydrogen bonds with EcR, one with each Asn390 and Glu199 having bond lengths 3.1 Å and 2.94 Å, respectively (Fig 1). From these results, it can be concluded that both these ligands form stable complexes with the target protein EcR.

**Table 1:** Binding free energy, number of H-bonds formed, amino acid residues involved in H-bonding, and bond lengths for the top 10 docked complexes.

S. No.	Ligand	Binding Energy (kcal/mol)	No. of H-Bonds	Amino acid residues involved in H-bonding	Bond Length (Å)
1.	ZINC08952607	-13.4	4	Thr231-O	2.84
				Thr231-O	2.92
				Thr231-O	2.57
				Ile227-O	3.18
2.	ZINC04264850	-13.4	2	Asn390-N	3.1
				Glu199-O	2.94
3.	ZINC03844966	-13.2	3	Ala286-N	3.0
				Thr393-O	3.08
				Thr304-O	2.83
4.	ZINC04027828	-13.1	3	Arg275-N	2.91
				Leu284-O	3.94
				Glu199-O	2.91
5.	ZINC08952608	-13.1	2	Thr231-O	2.95
				Thr231-O	2.77
6.	ZINC68589458	-13.0	3	Thr234-O	2.9
				Thr234-O	2.83
				Thr231-O	2.99
7.	ZINC08878289	-13.0	3	Thr234-O	2.91
				Thr234-O	2.79
				Thr231-O	3.03
8.	ZINC33927923	-13.0	2	Thr393-O	2.99
				Thr304-O	3.1
9.	ZINC04082366	-13.0	1	Asn390-N	3.04
10.	ZINC19938609	-12.9	3	Asn390-N	2.99
				Thr231-O	2.71
				Ala283-N	3.26

### 3.4. Calculation and analysis of physicochemical properties

**Fig 2:**
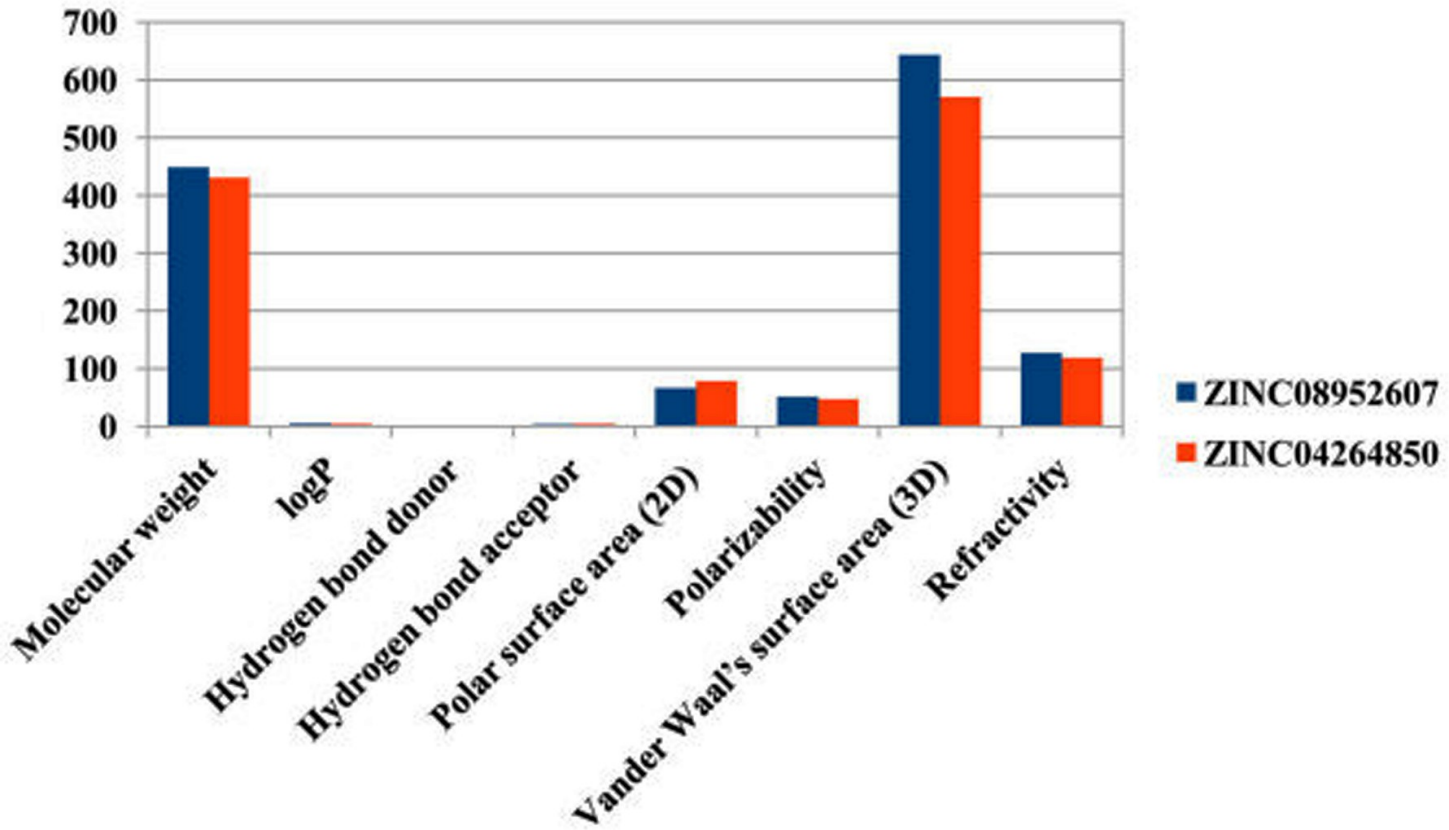
Values of principal descriptors for ZINC08952607 and ZINC04264850.

A drug should have a molecular weight less than 500 daltons, logP value less than 5, hydrogen bond donors less than 5, and hydrogen bond acceptors less than 10 [33]. The physicochemical parameter (like molecular weight, logP, H-bond donor and H-Bond acceptor) and other properties like polar surface area (2D), polarizability, van der Waal’s surface area (3D), and refractivity was considered according to other agrochemicals [34, 35]. The top two ligands ZINC08952607 and ZINC04264850 have molecular weight 448.607 and 430.614, logP value 4.97 and 4.253, number of Hydrogen-bond donors 2 and 2, and number of hydrogen-bond acceptors 3 and 4, respectively. Also, the polar surface area less than 140 Å is an indication of good cell membrane permeability of a drug and refractivity ranging from 40 to 130 indicates a better drug or agrochemical properties. ZINC08952607 and ZINC04264850 show polar surface area of 66.65 and 78.76, Polarizability 51.55 and 47.10, Van der Waal’s surface area 643.52 and 570.78, and refractivity 127.34 and 118.55, respectively (Table 2). These results show that both the ligands ZINC08952607 and ZINC04264850 possess the physico-chemical properties of a good agrochemical and can be used as a potential insecticide candidate against whitefly and other related insects (Fig 2).

**Table 2:** Physicochemical properties i.e. molecular weight, LogP, hydrogen bond donor, hydrogen bond acceptor, polar surface area (2D), polarizability, Van der Waal’s surface area (3D), and refractivity of top 10 ligands.

S. No.	Ligand	Molecular weight	log P	H-bond donor	H-bond acceptor	Polar surface area (2D)(Å)	Polarizability	Van der Waal’s surface area (3D)(Å)	Refractivity
1.	ZINC08952607	448.607	4.97	2	3	66.65	51.55	643.52	127.34
2.	ZINC04264850	430.614	4.253	2	4	78.76	47.10	570.78	118.55
3.	ZINC03844966	418.368	3.085	0	6	139.22	41.70	498.20	134.59
4.	ZINC04027828	396.502	4.593	2	3	49.69	43.08	551.83	110.58
5.	ZINC08952608	448.607	4.97	2	3	66.65	51.55	652.86	127.34
6.	ZINC68589458	408.457	3.639	0	3	57.69	47.28	519.18	117.16
7.	ZINC08878289	448.519	3.293	0	6	76.15	47.37	569.44	76.15
8.	ZINC33927923	391.559	5.376	1	3	54.35	44.83	614.96	116.69
9.	ZINC04082366	429.645	5.386	1	3	46.61	49.18	683.76	123.23
10.	ZINC19938609	407.466	4.311	1	5	68.23	43.86	548.94	113.78

### 3.5. Molecular dynamics simulations for evaluating the stability and conformational dynamics

All atom MDS is a technique that allows us to unravel the structural dynamics, conformational behavior, and stability of the protein and protein-ligand complexes. The field of structure-based drug discovery has been revolutionized with the advent of MDS. To understand the dynamic perturbations taking place in BtEcR during ligand binding, the MDS study was conducted. It also enabled us to predict the stability of the protein-ligand complexes (BtEcR-ZINC08952607, and BtEcR- ZINC04264850). In this study, RMSD, RMSF, Rg, and hydrogen bonds of the protein-ligand complexes were calculated. Also, the principal component analysis and binding free energy analysis were also performed. RMSD analysis revealed that the entire trajectory was stabilized after 30 ns. Therefore, all the parameters mentioned above were calculated for the last 20ns trajectory.

#### 3.5.1. Root Mean Square Deviation (RMSD)

**Fig 3:**
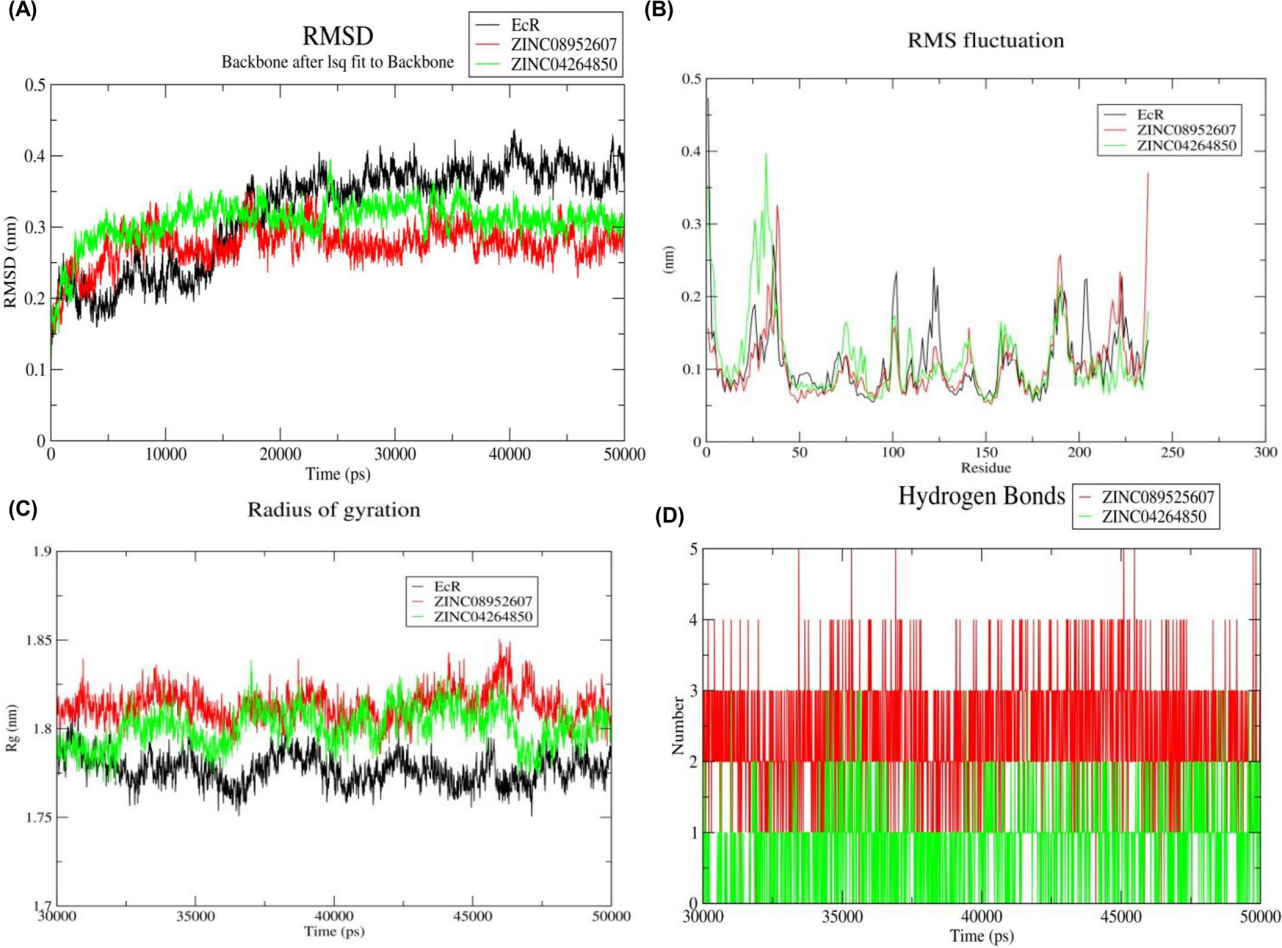
**(A)** Time dependent RMSD of c-α backbone of the BtEcR, BtEcR-ZINC08952607, and BtEcR-ZINC04264850. **(B)** The RMSF for c-α atoms of BtEcR, BtEcR-ZINC08952607, and BtEcR-ZINC04264850. **(C)** Plot of Rg vs time for BtEcR, BtEcR-ZINC08952607, and BtEcR-ZINC04264850. **(D)** Plot of number of hydrogen bonds vs time for the BtEcR-ZINC08952607, and BtEcR-ZINC04264850. Black, red and green color represents BtEcR, BtEcR-ZINC08952607, and BtEcR-ZINC04264850, respectively.

RMSD is the measure of the deviation of the protein backbone from its initial structural conformation to its final conformation. Deviations produced throughout the simulation determine the stability of the protein concerning its structural conformation. A stable protein structure shows smaller deviations in the protein backbone and vice versa. RMSD value for the Cα backbone of all three systems was calculated for the last 20ns simulation. Fig 3A shows the plot of RMSD (nm) vs. time (ps) for BtEcR, BtEcR-ZINC08952607, and BtEcR-ZINC04264850. The average RMSD values for BtEcR, BtEcR-ZINC08952607, and BtEcR-ZINC04264850 were found to 0.376, 0.281, and 0.315 nm respectively. Both, BtEcR-ZINC08952607 and BtEcR-ZINC04264850 showed lower RMSD value as compared to BtEcR, suggesting greater stability of BtEcR-ligand complexes than BtEcR.

#### 3.5.2. Root Mean Square Fluctuation (RMSF)

RMSF analysis describes the flexible regions of the protein-ligand complexes. In proteins, higher RMSF is shown by loosely organized structures like loops, turns, and coils while well-structured regions such as α-helix and β-sheets show low RMS fluctuation. We have calculated the RMSF value for prophesying the structural changes induced in protein structure by ligand binding. Fig 3B shows the RMSF plots for BtEcR, BtEcR-ZINC08952607, and BtEcR-ZINC04264850. The average RMSF value for BtEcR, BtEcR-ZINC08952607, and BtEcR-ZINC04264850 were found 0.109, 0.104, and 0.114 nm respectively. BtEcR-ZINC08952607 showed less RMSF value than BtEcR, which implies that upon ZINC08952607 ligand binding, the flexibility of catalytically significant residues of BtEcR has reduced. Hence, it can be concluded that ZINC08953607 can act as a potential inhibitor of BtEcR.

#### 3.5.3. Radius of gyration (Rg)

The radius of gyration indicates the compactness level of the protein structure before and after the binding of the ligand. Fig 3C shows the Rg plot for BtEcR, BtEcR-ZINC08952607 and BtEcR-ZINC04264850 with average Rg values as 1.777, 1.813, 1.800 nm, respectively. BtEcR-ZINC04264850 complex showed slightly lesser Rg as compared to the other complex, indicating that on binding with BtEcR, ZINC04264850 may form a relatively more stable complex as compared to ZINC08952607.

#### 3.5.4. Hydrogen bonds analysis

The hydrogen bond interactions are transitory and play an important role in the stabilization of protein-ligand complex. Fig 3D shows the H-bonding plot for BtEcR-ZINC08952607, and BtEcR-ZINC04264850. The complex BtEcR-ZINC08952607 formed an average of 0–5 hydrogen bonds while complex BtEcR-ZINC04264850 formed an average of 0–3 hydrogen bonds. The overall result of hydrogen bonds indicates that both the ligands can form stable complexes with the BtEcR protein. The ligand ZINC0852607 forms more H-bonds with BtEcR, indicating that it forms a more stable complex.

#### 3.5.5. Principal Component Analysis (PCA)

**Fig 4:**
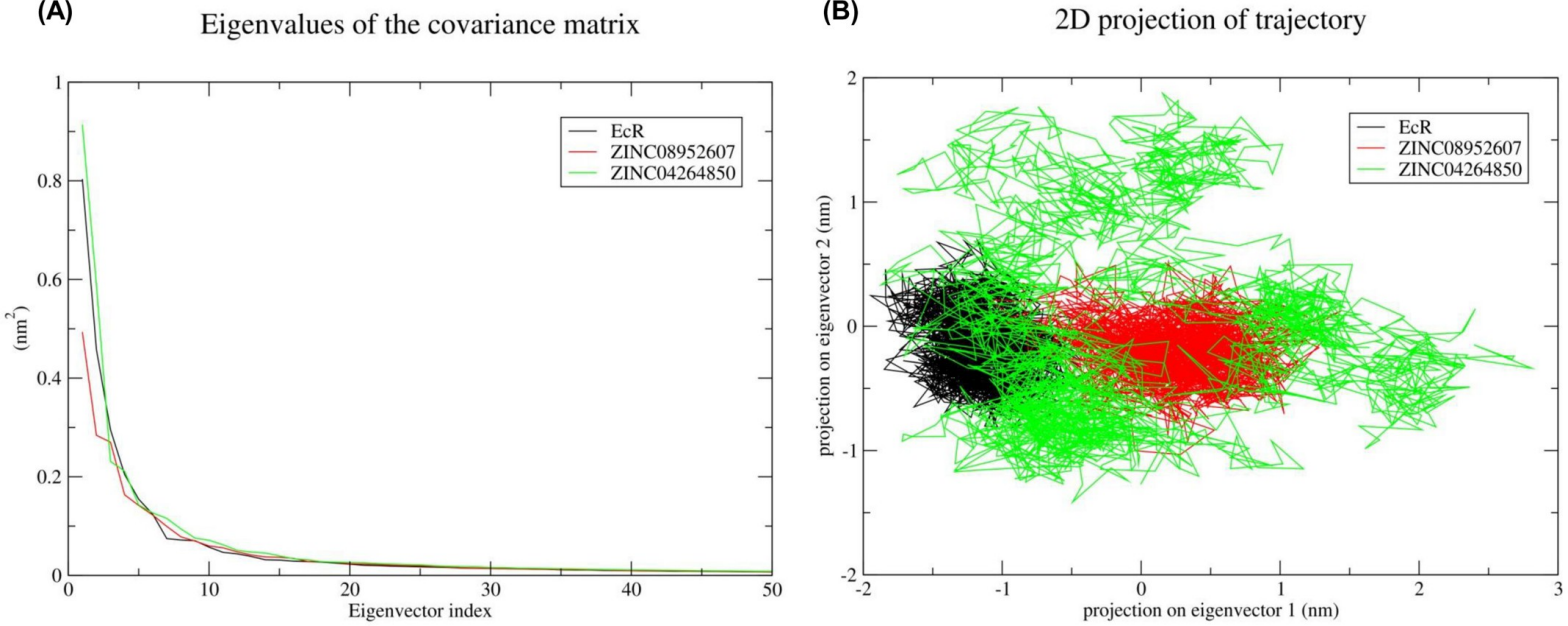
Principal component analysis. **(A)** Plot of eigen values vs eigenvector index was plotted taking into consideration the first 50 eigen vectors for BtEcR, BtEcR-ZINC08952607, and BtEcR-ZINC04264850. **(B)** Projection of protein motion in phase space along PC1 and PC2 for BtEcR, BtEcR-ZINC08952607, and BtEcR-ZINC04264850. Black, red and green color represent BtEcR, BtEcR-ZINC08952607 and BtEcR-ZINC04264850 complex, respectively.

**Fig 5:**
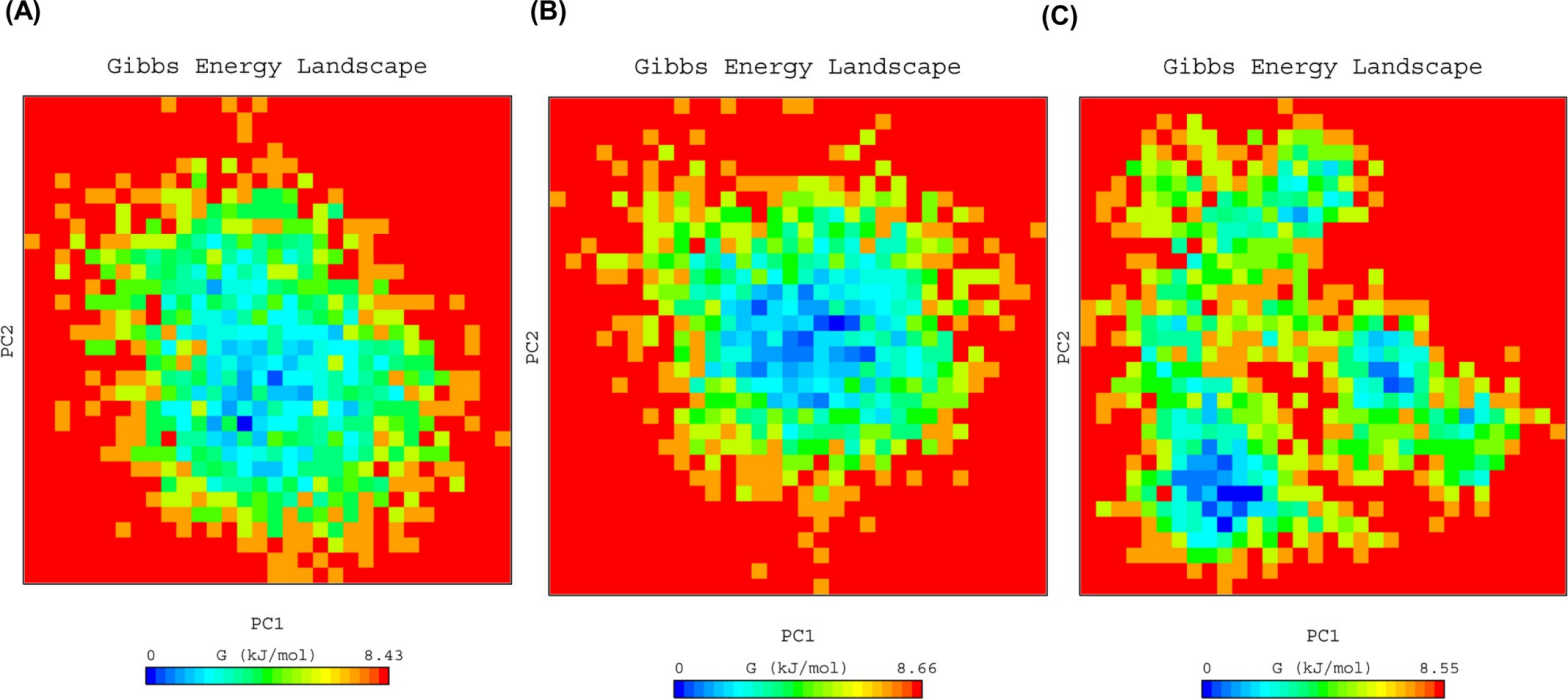
PC1 and PC2 Gibbs free energy landscape for **(A)** BtEcR, **(B)** BtEcR-ZINC08952607, and **(C)** BtEcR-ZINC04264850.

PCA was carried out for the prediction of large concerted motions during ligand binding. It is known that the first few eigenvectors describe the overall motions of the protein. So we used the first 50 eigenvectors to calculate the significantly correlated motions for the last 20 ns simulations. Eigenvalues were calculated after diagonalisation of the covariance matrix of atomic fluctuations. Fig 4A shows the plot of eigenvalues in descending order vs. the corresponding eigenvector for BtEcR, BtEcR-ZINC08952607, and BtEcR-ZINC04264850. The first 10 eigenvectors exhibit 77.06, 70.85 and 75.67 percent motions for BtEcR, BtEcR-ZINC08952607 and BtEcR-ZINC04264850, respectively. This indicates that the protein and protein-ligand complexes show different proportions of motions. Hence, it was inferred that binding of the ligand causes change in the protein conformation and dynamics. BtEcR-ligand complexes show lesser correlated motions as compared to BtEcR, which implies that ligand binding leads to complex stabilization. From the results, it was concluded that the BtEcR-ZINC08952607 complex is exhibiting lesser correlated motions and is more stable than BtEcR-ZINC04264850. From Fig 4A, it is clear that the first few eigenvectors play an important role in overall motions. So we used the first two eigenvectors for prediction of 2D projection plots for better representations of the results. The 2D projection plot representing the protein motions in phase space was plotted for BtEcR, BtEcR-ZINC08952607, and BtEcR-ZINC04264850 (Fig 4B). From the plot, it is evident that EcR-ZINC08952607 forms a more stable cluster as compared to BtEcR-ZINC04264850. Fig 5 shows the Gibbs free energy for PC1 and PC2. The plot shows energy ranging between 0 to 8.43, 0 to 8.66, and 0 to 8.55 kJ/mol for BtEcR, BtEcR-ZINC08952607, and BtEcR-ZINC04264850, respectively. BtEcR-ZINC04264850 complex showed lesser Gibbs free energy as compared to BtEcR-ZINC08952607, which implies that the complex followed an energetically more favourable transition from one conformation to another. The BtEcR-ZINC04264850 complex showed a higher blue-colored region indicating lower energy, and hence suggesting that this complex is thermodynamically more stable.

#### 3.5.6. MM-PBSA: Calculation and analysis of binding free energy

Binding free energy is the total of all the non-bonded interactions. It was estimated for BtEcR-ZINC08952607 and BtEcR-ZINC04264850, using MM-PBSA method. The interaction energies like, Van der Waal’s energy, polar solvation energy, electrostatic energy, SASA energy, and binding energy were calculated for last 5 ns of MD trajectory and are tabulated in Table 3. The binding free energy for BtEcR-ZINC08952607 and BtEcR-ZINC04264850 is −170.156 kJ mol^-1^ and −200.349 kJmol^-1^, respectively. From the results, it is evident that these compounds show a significant binding affinity with BtEcR, but ZINC04264850 shows a higher binding affinity as compared to ZINC08952607.

**Table 3:** Van der Waal’s, electrostatic, polar salvation, SASA and binding energy in kJ/mol for top two complexes.

Sr. No.	Compound	Van der waal’s energy (kJ mol^-1^)	Electrostatic energy (kJ mol^-1^)	Polar solvation energy (kJ mol^-1^)	SASA energy (kJ mol^-1^)	Binding energy (kJ mol^-1^)
1	ZINC 08952607	-247.973 +/- 11.225	-26.227 +/- 5.427	127.589 +/- 9.382	-23.545 +/- 1.049	-170.156 +/- 12.658
2	ZINC04264850	-241.724 +/- 10.213	-13.819 +/- 5.831	78.048 +/- 7.531	-22.854 +/- 1.061	-200.349 +/- 10.772

## 4. Conclusion

Cotton whitefly (*Bemisia tabaci*) is a highly destructive polyphagous pest that causes huge economic losses, inter alia in cotton by direct feeding and lint contamination. The repeated use of a limited number of effective chemical insecticides would results in insecticide resistance, limiting their use over a longer time. In the current study, a series of *in-silico* approaches were used to search some efficient natural lead that can target BtEcR. This computational study indicates that ZINC08952607 and ZINC04264850 have the potential to be developed as natural insecticides against *B*. *tabaci*. In future, activity of both these compounds can be optimized for much better potential, and insecticidal activity of potential inhibitors against *B*. *tabaci* can be tested in the laboratory and field conditions for crop protection.

## Supporting information

S1 File (DOC)
