# Supplementary material for: Virtual screening, molecular dynamics and binding energy-MM-PBSA studies of natural compounds to identify potential EcR inhibitors against Bemisia tabaci Gennadius
Source: PLoS One. 2022 Jan 21;17(1):e0261545. doi: 10.1371/journal.pone.0261545 (PMC8782374; doi:10.1371/journal.pone.0261545)
Supplement: S1 File — (DOC) [file pone.0261545.s001.doc]

All the script is available at <https://vina.scripps.edu/manual/>
